# Design and test of intelligent inspection and monitoring system for cotton bale storage based on RFID

**DOI:** 10.1038/s41598-022-08229-6

**Published:** 2022-03-16

**Authors:** Weipeng Zhang, Bo Zhao, Qizhi Yang, Liming Zhou, Hanlu Jiang, Kang Niu, Jian Ding

**Affiliations:** 1 The State Key Laboratory of Soil-Plant-Machinery System Technology, Chinese Academy of Agricultural Mechanization Sciences, Beijing, 100083 China; 2 Jiangsu University, Zhenjiang, China; 3 Agricultural Machinery Service Center in Pingdu City, Pingdu, 266700 China

**Keywords:** Electrical and electronic engineering, Mechanical engineering

## Abstract

To solve the inspection problems in cotton storage, as well as the need for environmental monitoring in the process of modern cotton bale storage, an intelligent inspection and temperature and humidity intelligent monitoring system based on RFID cotton bale was developed by adopting RFID (Radio Frequency Identification) technology, wireless temperature and humidity real-time monitoring technology and handheld terminal intelligent inspection technology. The system was composed of RFID positioning inspection module and temperature and humidity real-time monitoring and transmission module. The artificial neural network (ANN) based on the particle swarm optimization (PSO) algorithm was used to process the monitoring data of the system by Gaussian filtering, and an accurate classification model of RSSI and label position was established. The test results showed that: Through the comparative analysis of the RFID indoor positioning algorithm, the positioning error of the PSO-ANN algorithm was small. In the actual cotton bale warehouse test, the relative error of positioning and monitoring for RFID cotton bale intelligent inspection and monitoring system was less than 6.7%, which effectively improved the working efficiency of inspection personnel and the security of cotton bale storage. The relative error of temperature and humidity was less than 8% and less than 7%, which could display the temperature and humidity information in real time and meet the real-time demand. This study improved the management personnel's effective positioning and inspection of the cotton bale, prevented the loss of cotton bale, reduced the deterioration probability of cotton bale, and effectively improved the storage management level of the cotton bale. It was of great practical significance to realize the networking, automation, and intelligence of cotton bale storage management.

## Introduction

The number and scale of warehousing enterprises in China are showing a trend of rapid growth. At the same time, due to national conditions and development reasons, as well as the physical characteristics and special storage environment of cotton, there are problems such as mildew, fire, theft, etc.^1–3^. For a long time, for the safety of cotton in the warehouse, bale warehouse still adopts the methods of manual periodic inspection, which requires a large number of inspectors to put into work. Due to the variability of manual inspection^4^, it is difficult to ensure the quality of decision management, especially the means and methods of large and medium-sized intelligent cotton storage urgently need to be improved. It is very important to carry out real-time inspection and temperature and humidity monitoring of cotton stacks in the process of cotton storage.

For the monitoring and processing of positioning information^5,6^, domestic and foreign experts and scholars have carried out research on multi-sensor fusion technology^7,8^, wireless communication technology^9–13^, and optimization algorithm technology^14–16^, which have effectively improved the intelligence level of critical operation monitoring^17^. Since RFID^18,19^ has the advantages of non-contact reading of information, large data storage capacity, and small volume^20–22^, it could be better applied in monitoring and management of cotton stack storage^23^, and the optimization processing algorithm was adopted to improve positioning^24,25^ and environmental temperature and humidity monitoring accuracy^26^. For example, Ullah et al.^27^ developed a mathematical model, compared with the cost of the card reader, the proposed RFID system has more significant sensing power Omer et al.^28^ developed a new procedure to estimate the indoor distance of passive UHF RFID tags, which effectively improved positioning monitoring accuracy. Srbinovska et al.^29^ designed a practical low-cost greenhouse monitoring system based on a wireless sensor network technology to monitor key environmental parameters such as temperature, humidity, lighting. Wu et al.^30^ proposed a new differential received signal strength (RSS) positioning algorithm, which can better achieve device tracking accuracy compared with the existing RFID positioning method. Badia-Melis et al.^31^ adopted WSN and RFID technology to realize temperature and humidity detection and positioning of the cold chain, which can monitor temperature and position changes at a high rate. According to the physical and chemical characteristics of cotton, JU^32^ proposed a set of fire prevention measures for cotton logistics warehouses, and obtained the optimal temperature and humidity threshold in the cotton storage environment. To sum up, the application of sensors has been effectively studied in various fields^33,34^, but the development and application of the existing storage monitoring system^35,36^ can only realize intelligent collection and management in the process of logistics and warehousing^37^ and the corresponding technologies^38,39^ have not been applied in the collection of cotton stack storage, it is imperative to develop a terminal for intelligent monitoring inspection of cotton bale.

To improve the management of cotton warehouse, this research broke through key technologies of real-time monitoring of cotton bag storage environment and artificial inspection positioning. Aiming at Cotton stack positioning and storage environment temperature and humidity monitoring, an RFID intelligent inspection system for cotton bag storage was developed, which realized the informationization and intelligence of cotton bale inventory management, and integration of host computer service platform and handheld terminal information. The system ran stably and reliably. The research results provided reference for related cotton bale storage.

## The overall structure and working principle of the system

### System structure design

**Figure 1:**
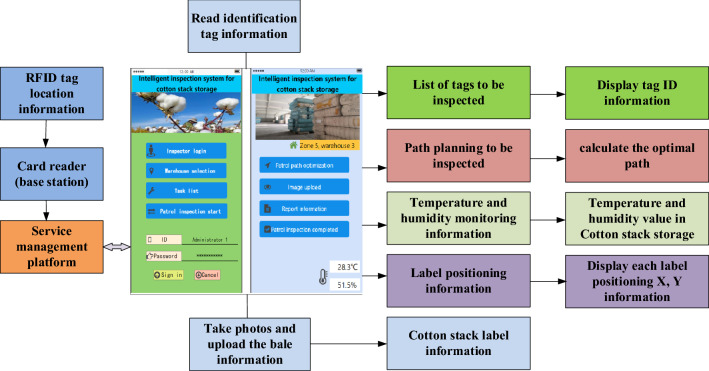
System structure design block diagram.

As shown in Fig. 1, RFID storage intelligent inspection and monitoring system is composed of location server, wireless card reader, network card reader, temperature and humidity sensor, handheld terminal, etc. The location server, as the upper terminal of the positioning system, is connected with the host platform to check the real-time RFID tag position. The location server calculates the specific location of the tag after comprehensive data calculation. The temperature and humidity sensor adopts Bluetooth wireless communication technology to realize the real-time data transmission of temperature and humidity monitoring. The handheld terminal can read and identify RFID tags, view inspection information, take photos and upload the information of cotton bags, etc. To read the identification label information, the terminal uses radio frequency identification (RFID) technology and code scanning technology to collect and upload the packet information to the platform during the inspection.

### Working principle of RFID intelligent inspection system

**Figure 2:**
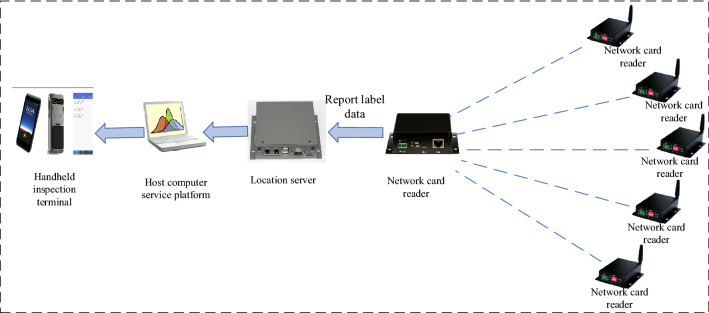
Schematic diagram of the working principle of the RFID intelligent inspection system.

As shown in Fig. 2, RFID technology is used as the basis to realize real-time collection of the information of the cotton stacks in the warehouse. RFID tags are affixed to the cotton stacks in the storage process. At the same time, the wireless card readers are orderly arranged in the warehouse, and the label information read by wireless card readers are reported to the network card reader. Path optimization is achieved by using the Greedy algorithm, and then the optimal path planning route is transmitted to the handheld terminal.

### Principle of temperature and humidity wireless monitoring

**Figure 3:**
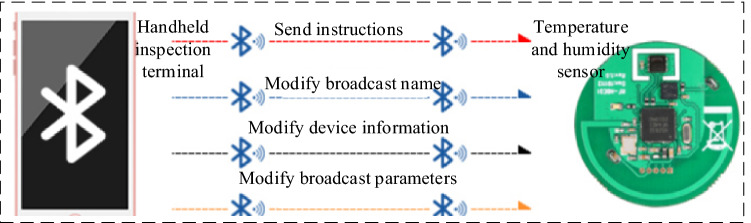
Schematic diagram of temperature and humidity sensor principle.

As shown in Fig. 3, the wireless monitoring of temperature and humidity mainly collects the cotton stack warehouse in real-time and with high precision. The temperature and humidity sensor is arranged in the cotton bale warehouse to send the temperature and humidity value of the cotton bale warehouse to the handheld terminal through wireless transmission.

## Hardware system design

**Figure 4:**
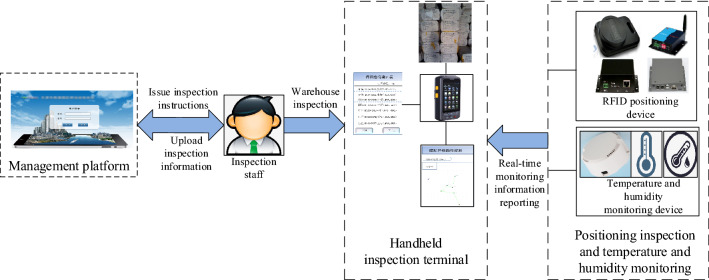
The overall structure of the hardware system.

As shown in Fig. 4, the system hardware is mainly composed of integrated temperature and humidity sensors, RFID positioning system devices, and intelligent inspection terminal equipment.

### RFID inspection module

**Figure 5:**
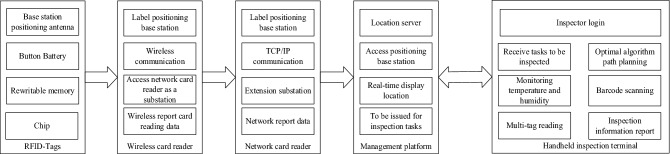
Block diagram of the hardware structure of the RFID inspection system.

As shown in Fig. 5, RFID positioning inspection is mainly composed of RFID tag, network card readers, wireless card reader, location service platform, and handheld terminal. RFID tag is an active electronic tag used to locate the cotton stack. Fixing the electronic tag on the cotton stack and installing a card reader around or on the top of the room, the real-time monitoring of the position of the indoor cotton stack can be realized and the specific position and distance of the electronic tag within the effective range can be determined.

### Location server

**Figure 6:**
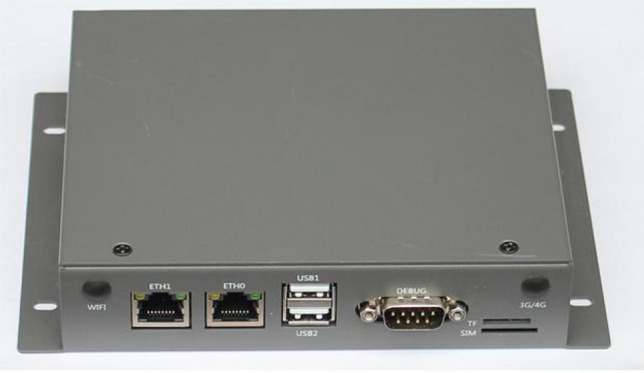
Location server.

The location server is shown in Fig. 6. Each positioning base station accesses to the location server and receives the label information from each positioning base station. Through data analysis and signal field strength calculation, the location information of each label is obtained and finally sent to the management platform, which sends the inspection information to the handheld terminal. The technical parameters are shown in Table 1.

**Table 1:** Technical parameters.

Project	Parameter
CPU	CPU
	RAM
	ETH0
Ethernet	ETH1
Debug	RS232
Size	160 mm × 105 mm × 33 mm
Power supply	DC12V

### Electronic label network card reader

**Figure 7:**
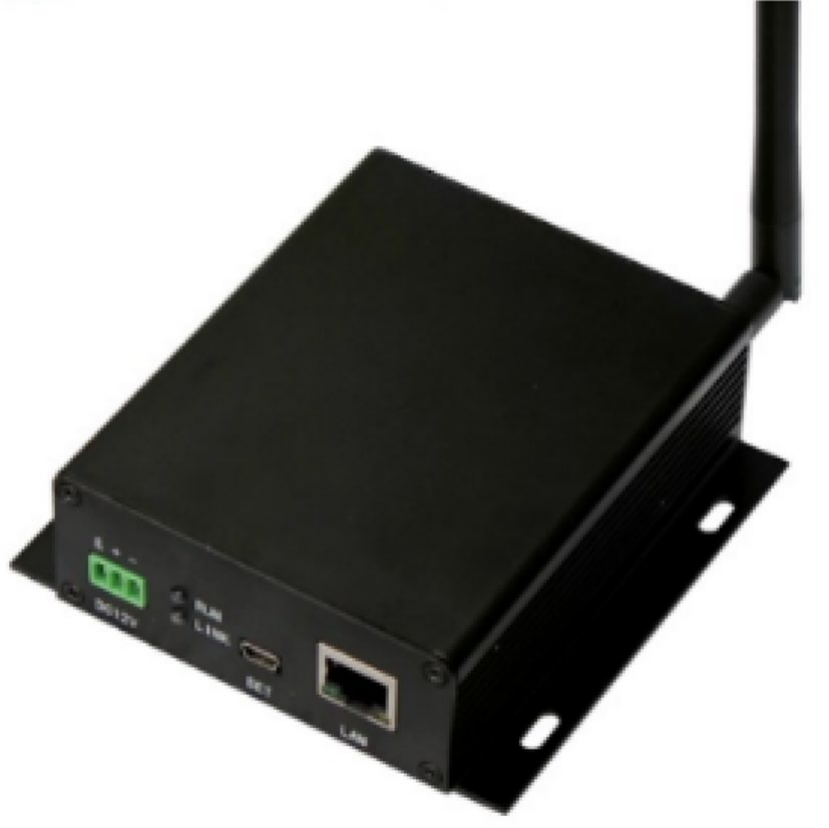
Electronic tag network card reader.

As shown in Fig. 7, it is a network card reader. The network card reader can access up to 16 wireless card readers, including itself (the device itself is also an independent card reader). A wireless network can have up to 17 A positioning base stations, which can quickly realize the deployment of the positioning base station in the cotton bale warehouse. The network card reader can be connected to the wireless card reader base station, and it can also be connected to a card reader antenna as a card reader base station. The network card reader reports the real-time received positioning information to the platform. As shown in Table 2, the card reader antenna can be extended to 50 m (9600 bps), can measure the field strength of the wireless signal of the electronic tag, and calculate the actual distance of the tag. The network card reader is with 10–26 V wide voltage power supply, and consumption current does not exceed 500 mA (12 V).

**Table 2:** Technical parameters.

Class	Parameter
Wireless network interface	Carrier frequency 433 MHz
	Transmit power 30 dBm
	Receiving sensitivity − 145 dBm
	Wireless rate 19,200 bps
	Reference communication distance 2000 m
Reader antenna interface	Communication interface RS232 serial port
	Communication baud rate 115,200, 57,600, 38,400, 19,200, 9600 bps
Working temperature and humidity	Interface specifications 4P terminal head
	− 40–85℃, humidity is less than 95%
Power supply	12 V(1A)
Size	82 mm × 103 mm × 33 mm

### Electronic tag wireless card reader

**Figure 8:**
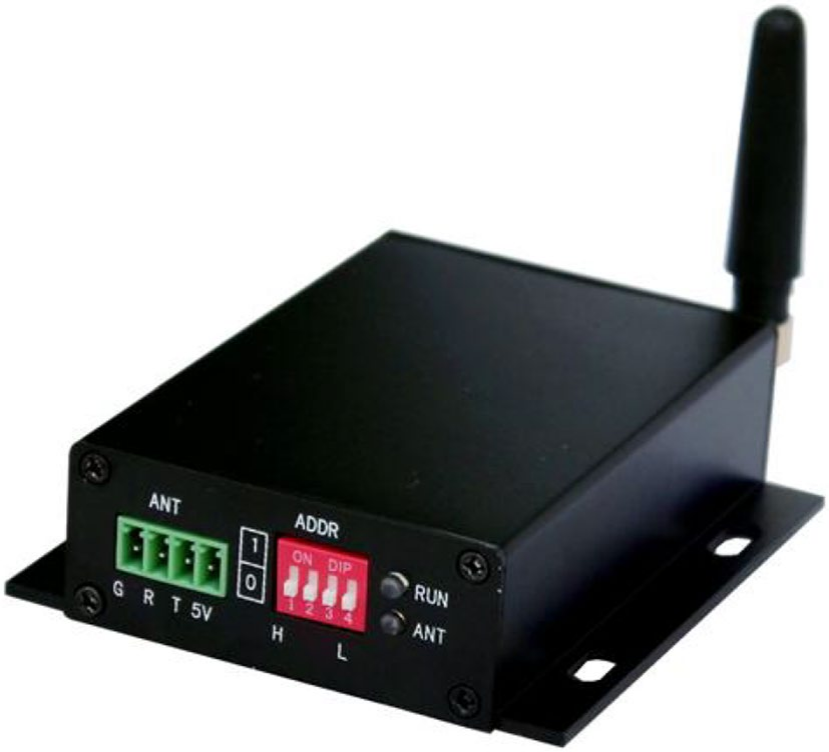
Wireless card reader.

As shown in Fig. 8, the wireless card reader is connected to the network card reader as a sub-station of the card reader. After reading the label information, the card reader reports the location information to the service platform. The specific location of the electronic tag within the effective range can be determined.

**Figure 9:**
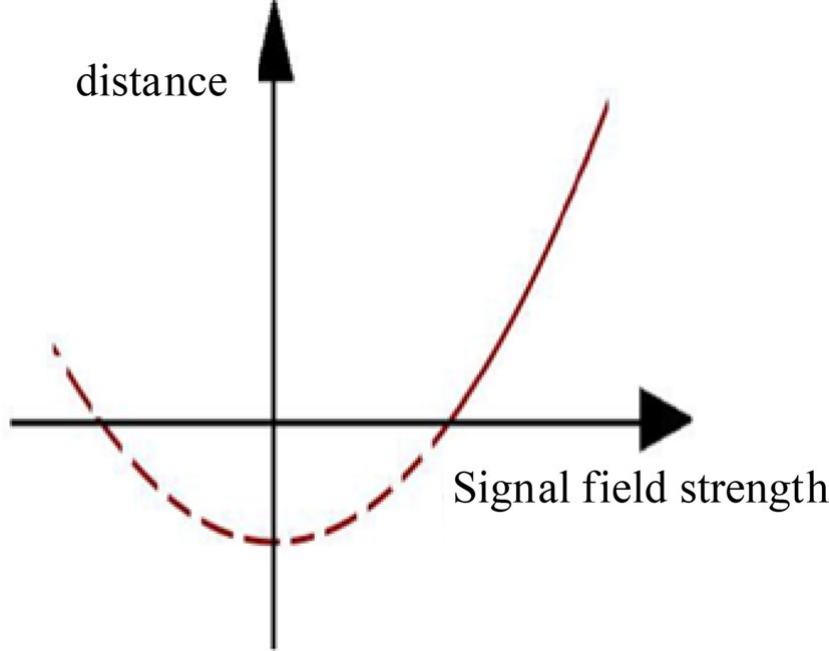
Parabolic graph of distance and wireless signal strength.

The distance between the electronic tag and the antenna is calculated by measuring the field strength of the tag wireless signal. The relationship between the distance and the wireless signal strength is parabolic. As shown in Fig. 9, the specific conversion formula of the distance measurement formula is:

1 $$D({\text{cm}}) = (RSSI({\text{dBm}}))^{2} \times P - L.$$

In the formula, D is the distance converted according to the signal strength, in cm; RSSI is the signal strength, and the absolute value is in dBm; P is the parameter, which is related to the antenna; L is the parameter, which is related to the antenna.

## The cotton stack positioning algorithm

ANN is a highly complex algorithm inspired by biology, which can solve complex nonlinear problems through modeling. Reference^40^ optimized the structure of ANN, determined the best vector network, improved the stability of the ANN algorithm, reduced the positioning error, but did not filter out the noise data in the experiment. Therefore, to improve the positioning accuracy, the data was processed by Gaussian filtering in this paper, and a positioning algorithm based on PSO optimization ANN(PSO-ANN) was proposed, and environmental noise was preprocessed by the Gaussian filtering to compensate for the positioning defects of ANN, thus improving the prediction effect of ANN and the learning speed of ANN.

### PSO-ANN positioning method

In this study, PSO was used to optimize the initial weight and threshold parameters of ANN, and Gaussian filtering was used to preprocess the noise barriers in the storage environment, which reduced the label positioning error and made up for the ANN positioning defect. In the real-time positioning of cotton bales, the signal strength will reflect, diffract and scatter in the storage environment and the common model of signal path loss in the storage environment is

2 $$RSSI(d) = P(d_{0} ) - 10n\lg \frac{d}{{d_{0} }} + X,$$

where RSSI(d) is the RSSI obtained by the reader, $P(d_{0} )$ is the RSSI with a reference distance of $d_{0}$, $n$ is the proportional coefficient of the path loss, and $X$ is the randomly distributed Gaussian noise.

PSO-ANN has outstanding performance in terms of efficiency and positioning accuracy. The PSO particle continuously updates its speed and position. The update formula is

3 $$\begin{aligned} V_{id} (i + 1) = & V_{id} (t){ + }c_{1} r_{1} (P_{best} (t) - X_{id} (t)) \\ & + c_{2} r_{2} (G_{best} (t) - X_{id} (t)), \\ \end{aligned}$$

4 $$X_{id} (t + 1) = X_{id} (t){ + }V_{id} (t + 1),$$

where $V_{id} (t)$ is the iterative velocity of the particle at the t time iteration,$X_{id} (t)$ is the iterative position of the particle, $c_{1}$ and $c_{2}$ are the learning factors, usually $c_{1}$ and $c_{2}$ have the same value, the value range is [1, 3], $r_{1}$ and $r_{2}$ are It is a uniform random number in the range of 0–1, $P_{best}$ is the best position of each particle during the update process, and $G_{best}$ is the value of all particles Best location.

### Cotton stack positioning model

**Figure 10:**
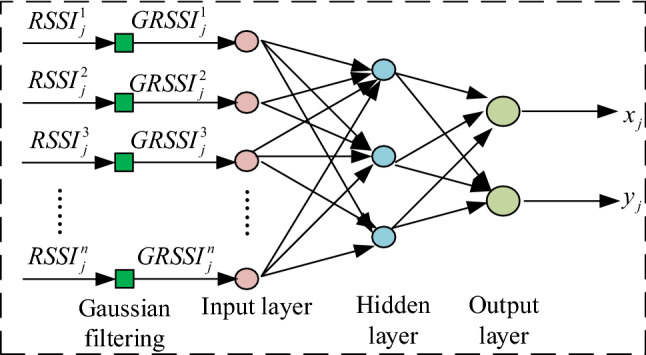
Structure of the positioning algorithm.

For cotton bale storage location, it is necessary to construct the mapping relationship between $RSSI$ and location (Loc) preprocessed by Gaussian filtering, that is,$GRSSI_{j} \to Loc_{j}$, where $GRSSI_{j}$ is the $RSSI$ value of the j time tag received by each reader, and $Loc_{j}$ is the first. The position coordinates of j labels. In the application process, $GRSSI_{j} = \{ GRSSI_{j}^{1} , GRSSI_{j}^{2} , GRSSI_{j}^{3} , \ldots GRSSI_{j}^{n} \}$; $Loc_{j}$ is the coordinates of the j-th reference label, that is $Loc_{j} = \{ x_{j} ,y_{j} \}$. Therefore, PSO-ANN was used in this paper to find the positioning target mapping connection $f:GRSSI_{j} \to Loc_{j}$ that is $f(GRSSI_{j} ) \approx Loc_{j}$. Since the above mapping relationship was non-linear, PSO-ANN was selected to solve this problem in the process of positioning the bale. The structure of the positioning algorithm is shown in Fig. 10.

### Gaussian filtering

To reduce the noise error, the collected data were processed by Gaussian filtering with the formula as follows:

5 $$f(RSSI) = \frac{1}{{\sigma \sqrt {2\pi } }}\exp \left[ { - \frac{{\left( {RSSI - A} \right)^{{2}} }}{{2\sigma^{2} }}} \right].$$

In the formula, σ^2^ is the variance, A is the expected value, and

6 $$\overline{\sigma } { = }\sqrt {\frac{1}{n - 1}\sum\limits_{i = 1}^{n} {(RSSI_{i} - \overline{A} )^{2} } } ,\,\overline{A} = \frac{1}{n}\sum\limits_{i = 1}^{n} {RSSI_{i} } ,$$

where n is the number of times to measure the RSSI value, and $RSSI_{j}$ is the RSSI value measured for the i-th time.

According to the 2$\overline{\sigma }$ Gaussian distribution criterion, remove the data with a small probability and easy to be disturbed, and keep the data as the effective date for the position positioning of the positioning system. This method can filter most erroneous data, so the accuracy of the system’s monitoring results is improved.

### Optimal layout of the network card reader

**Figure 11:**
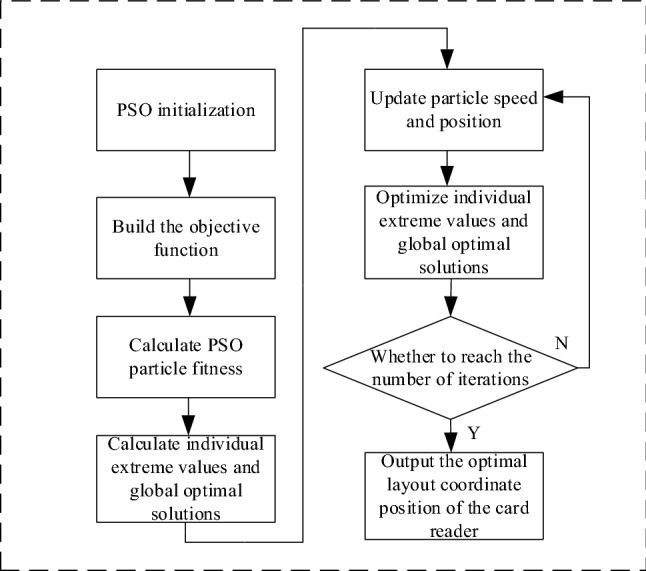
PSO optimizes the flow chart of reader position distribution.

The signal sent by the RFID reader to the tag decreases with the increase of the propagation distance. Since the RSSI value measured at a certain point during positioning will fluctuate constantly, and the fluctuation of the RSSI value generally follows the Gaussian distribution, the positioning result is related to the Euclidean distance of the signal space between the positioning tags. The layout of the card reader during storage can be equivalent to plane optimization, and the position of the reader on the plane distribution can be optimally solved by PSO algorithm. The solution process is shown in Fig. 11.

The specific steps of PSO-ANN are as follows:

1. Set the relevant parameters according to the actual input samples of the ANN: the number of nodes in the input layer is set to n, the number of nodes in the hidden layer is set to s, and the number of nodes in the output layer is set to m.
2. Set the PSO-related parameters: the initial position of the particles is $X_{0}$, the initial velocity is set to $V_{0}$, the number of particles is N, the maximum number of iterations is m, the learning factors are $c_{1}$ and $c_{2}$, and the values are equal.
3. Calculate the fitness value of each solution according to formula (7), and obtain $P_{best}$ and $G_{best}$.
  7 $$f = \frac{1}{N}\sum\limits_{i = 1}^{N} {\sum\limits_{j = 1}^{L} {\mathop {(Y_{ij} - y_{ij} )}\nolimits^{2} } } ,$$
  where N is the total number of training samples, L is the number of output neurons in the network, $y_{ij}$ is the expected output value of the i-th output node, and $y_{ij}$ is the actual output of the i-th output point.
4. Output the optimal global solution of the PSO algorithm, and calculate the current fitness value fi of all particles. If $f_{i} < P_{best}$, $P_{best}$ is the individual optimal, $X_{i} = P_{best}$, otherwise $f_{i}$ is the individual optimal. If $f_{i} < G_{best}$, then $G_{best}$ is the global optimal solution, $X_{i} = P_{best}$, Otherwise, $f_{i}$ is the optimal solution, where $X_{i}$ is the current position of the particle.
  The optimal solution, where $X_{i}$ is the current position of the particle.
5. According to Eqs. (3) and (4), update the particle velocity and position.
6. Determine whether to terminate: If the number of iterations reaches the expected specified value, terminate the process and output the optimal solution (the weight and threshold of the algorithm), otherwise, return to step (3).

### Temperature and humidity sensor

**Figure 12:**
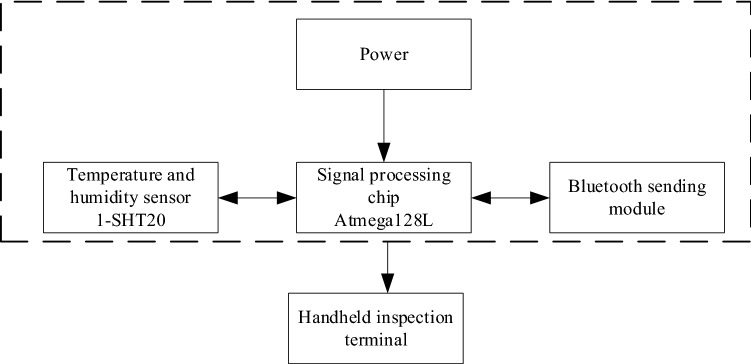
Block diagram of temperature and humidity sensor.

As shown in Fig. 12, it is a block diagram of the temperature and humidity sensor. To monitor the temperature and humidity in the cotton bale warehouse, a wireless temperature and humidity monitoring module, the sensor chip 1-SHT20 temperature and humidity sensor and sensor chip 2-LIS3DH three-axis acceleration sensor were selected in the system, with battery model CR2032, transmit power − 20 to + 4 dBm. The 1-s broadcast interval can work continuously for 1 year. The default output is 0 dBm, and the transmission distance is 50 m. ThenRF52832 wireless Bluetooth transmission technology is used to monitor temperature and humidity, and the maximum rate supports 400 kbps; size: 48 mm in diameter, and 23 mm in height.

Through the design of the temperature and humidity module of the RFID cotton bale intelligent inspection system, the temperature and humidity sensor was redeveloped and designed, in which the temperature conversion formula is:

8 $$T = d_{1} + d_{2} S_{oT} ,$$

where T is the temperature value, ℃; S_oT_ is the temperature data read from SHT20 by the main control chip; d_1_ and d_2_ are temperature conversion coefficients. Relative humidity conversion formula:

9 $$RH_{{{\text{linear}}}} { = }c_{{1}} { + }c_{{2}} S_{oRH} { + }c_{{3}} S_{oRH}^{2} ,$$

where RH_linear_ is the relative humidity; The humidity data read by S_oRH_ from 1-SHT20 by the main control chip; c_1_, c_2_ and c_3_ are humidity conversion coefficients. Because the measurement of humidity is affected by temperature, the temperature compensation of the sensor should also be considered when measuring humidity. The compensation formula is:

10 $$RH_{true} = (T - 25)(t_{1} + t_{2} S_{oRH} ) + RH_{linear} ,$$

where RH_true_ is absolute humidity; t_1_, t_2_ are temperature compensation coefficients. Combining the design of the temperature and humidity module, the temperature accuracy S_oT_ is 14 bits, and the humidity accuracy S_oRH_ is 12 bits. Substituting the coefficient results into Eqs. (8)–(10), we can get:

11 $$\left\{ \begin{array}{*{20}l} T = 39.66 + 0.11S_{oT} \hfill \\ RH_{linear} = - \;4 + 0.0405S_{oRH} - 2.8 \times 10^{ - 6} S_{oRH}^{2} \hfill \\ RH_{true} = \left( {T - 25} \right)\left( {0.01 + 0.000085_{oRH} } \right) + RH_{linear} \hfill \\ \end{array} \right..$$

## Software system design

**Figure 13:**
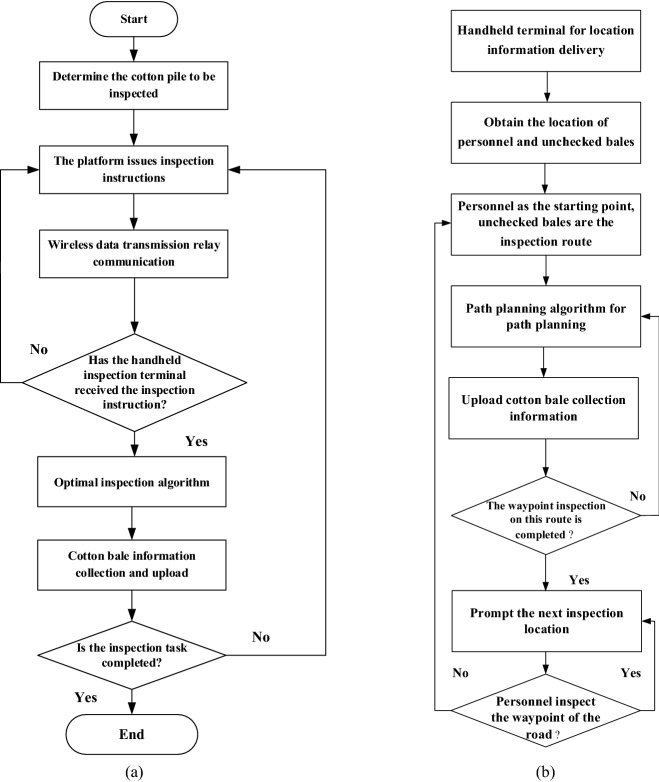
Software system flow chart. (**a**) Hand-held inspection terminal system software system design flowchart (**b**) RFID assisted manual inspection path planning process.

According to the requirements of reliability, real-time and maintainability of the cotton bale storage intelligent inspection monitoring system, the handheld inspection terminal system software program is written in Java language. The system software design flowchart is shown in Fig. 13a. When the system was started, the initialization program was first performed. After receiving the instruction to be inspected from the platform, the handheld terminal sent the position information of the cotton pile to be inspected to the handheld terminal device through wireless data transmission, and the inspection personnel collected the information of the cotton stack to be inspected to the handheld inspection terminal equipment through the handheld inspection terminal device. The inspection content included image collection of cotton stack, the barcode information of cotton stack, and temperature and humidity, which were uploaded to the platform system by taking pictures. As shown in Fig. 13b in the process of inspection, the handheld terminal device receives the inspection information of the platform, and adopts the greedy algorithm to realize the path optimization. taking The algorithm takes the position of the inspector as the starting point, and indicates the inspection route through signs to realize the optimal path optimization of the inspection personnel., and the algorithm is easy to implement and efficient.

## Test and analysis

### Simulation comparative analysis

**Figure 14:**
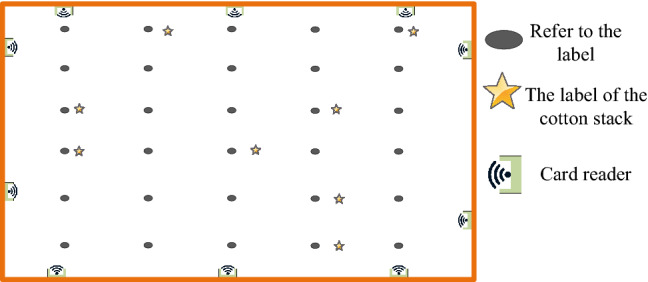
Location simulation environment schematic diagram.

To verify the feasibility of the RFID cotton bale intelligent inspection system, the test area is 25 m × 25 m, and the simulation environment is shown in Fig. 14. In the positioning map, the lower-left corner was the origin of coordinates, with 30 reference labels, and 10 reader antennas were placed in the positioning map to collect RSSI sample values of PSO-ANN. The distances to 10 readers were calculated respectively, and the corresponding RSSI vectors were generated according to Formula (1). The coordinates of the reference tag and its corresponding signal strength vectors were used as the training data of ANN and PSO-ANN so that the trained ANN and PSO-ANN could predict the position of the tag to be fixed. The RSSI vector of the reference label was used as the training data of PSO-ANN and ANN, and used as the input of the positioning model to predict the position coordinates of the cotton rick label. The network was trained by DPS. Through many experiments and simulations, the number of nodes in the hidden layer was 5, 3 of which was hidden layers, and the number of node in the hidden layer was 5. The position coordinates of the cotton stack were neurons of the output layer and were dependent variables, the excitation function was Sigmoid, the parameter was 0.9, the minimum training rate was 0.1, the allowable error was 0.0001, and the dynamic parameter was 0.6.

**Figure 15:**
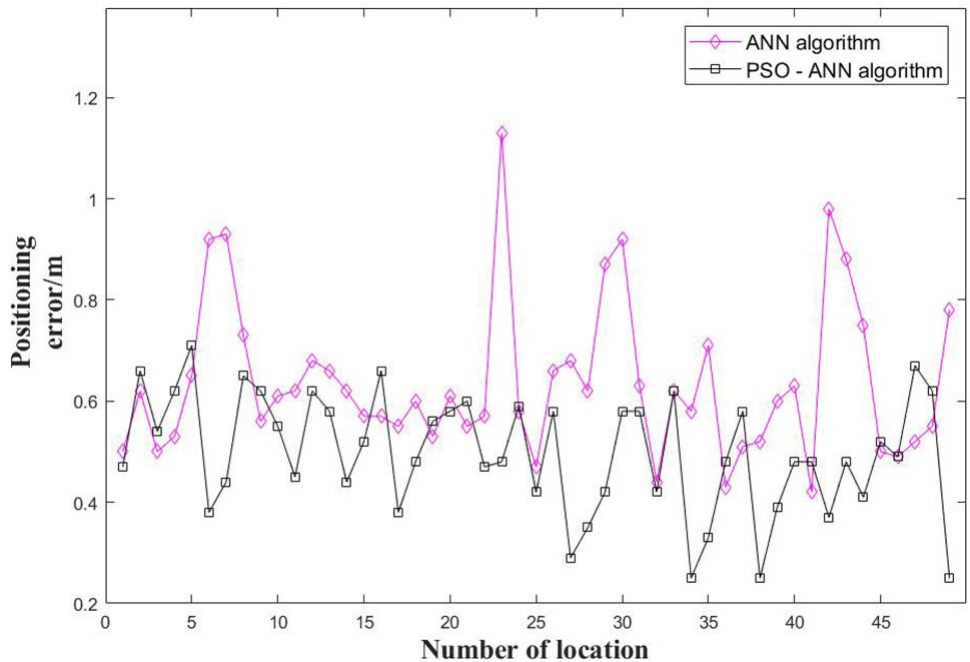
Error comparison of the two positioning algorithms.

This paper, the positioning errors of ANN and PSO-ANN were compared, and the comparison results are shown in Fig. 15. It can be seen from Fig. 15 that the positioning error of the PSO-ANN algorithm was generally smaller than that of the ANN algorithm, that is, PSO-ANN had better positioning accuracy of the cotton stack.

**Figure 16:**
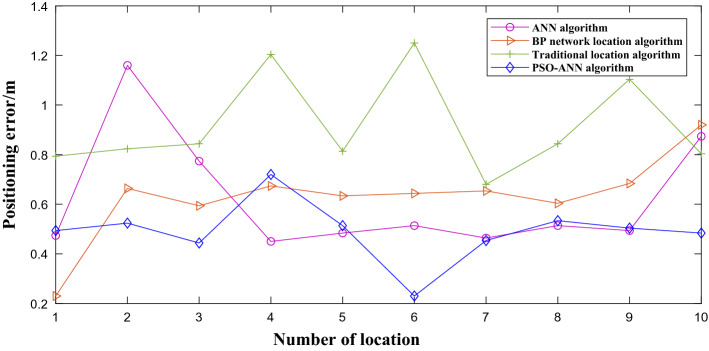
The legend of Table 3.

Table 3 shows the other two different localization algorithm the average position error obtained in reference^41^, by comparing the average error of 4 kinds of algorithms. It can be seen that the traditional algorithm positioning effect was inferior to the other three kinds of localization algorithm, and the proposed algorithm error was smaller than the reference^41^ of the algorithm, it shows that the proposed algorithm can provide relatively reliable positioning data (Fig. 16).

**Table 3:** Comparison of location results of the four algorithms.

Error/m	Traditional location algorithm	BP network location	ANN	PSO-ANN
Minimum error	0.68	0.23	0.45	0.23
Maximum error	1.25	0.92	1.16	0.72
The average error	0.97	0.65	0.62	0.49

ANN was easy to fall into local optimum in nonlinear prediction. Optimizing ANN with PSO can greatly improve the learning rate of ANN and find the global optimum solution through iteration. Table 4 compares the effects of different training methods on training efficiency and error. It can be seen from the results in Table 4 that the algorithm proposed in this paper can effectively reduce the training time and improve the positioning accuracy (Table 4).

**Table 4:** Training time comparison.

Method	ANN	PSO-ANN
Average error /m	0.62	0.49
Training time /s	5.42	1.21

### RFID cotton bale positioning test

**Figure 17:**
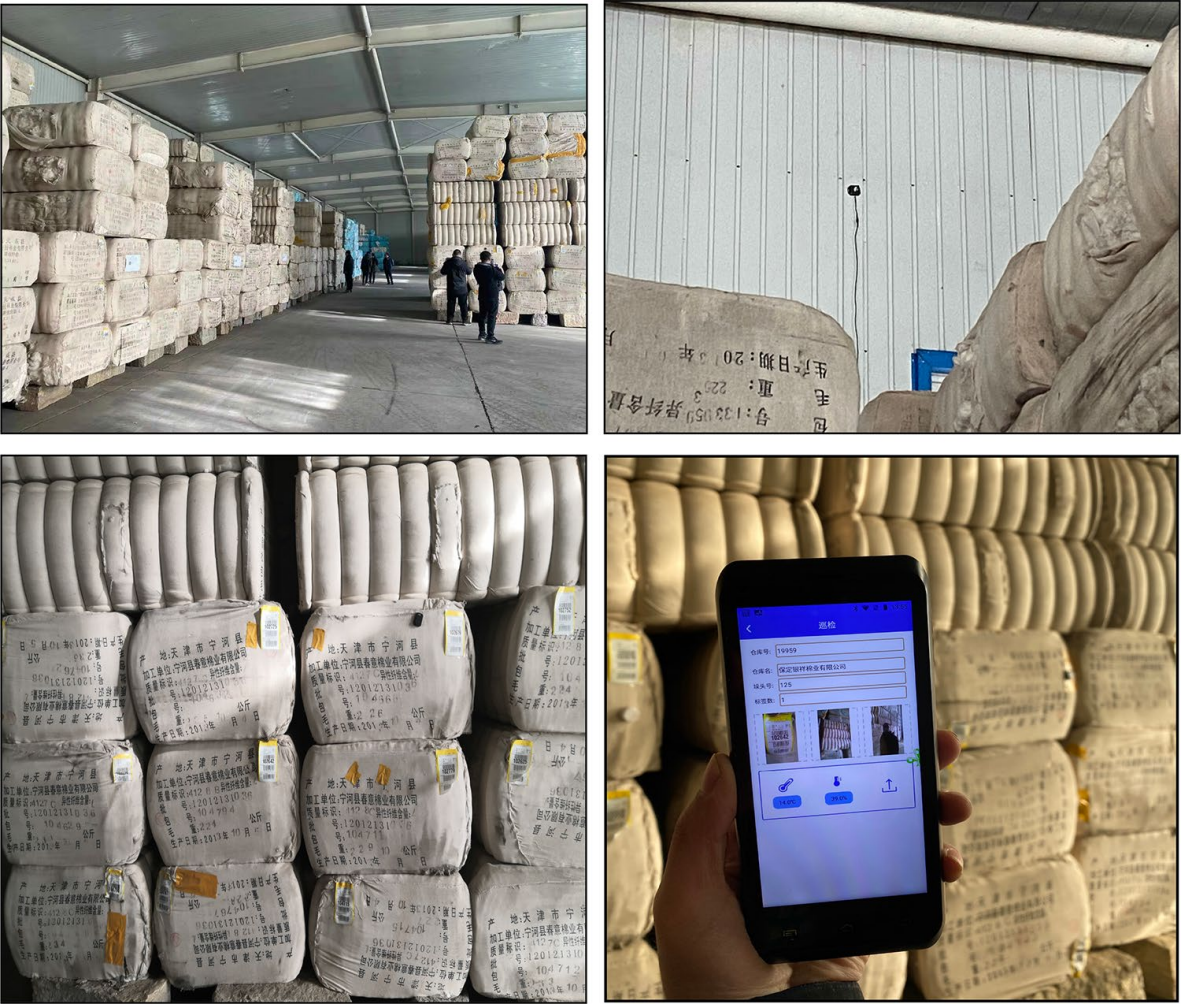
Field test of bale positioning inspection.

To further verify the practical application effect of RFID positioning inspection system in cotton bag warehouse, a performance test was carried out in December 2020 in Baoding in YIN XIANG cotton industry co., LTD., Hebei province. Figure 17 shows the bale warehouse test site. The cotton package storage storehouse was set up with 10 card reader, and Cotton stack RFID and wireless temperature and humidity sensor were placed in the cotton pile. The RFID tag was placed cotton bags to start the location service platform, and the location server, network card reader, and wireless card reader device were powered on, which can effectively monitor the RFID tag and real-time movement, and display the coordinate position information of the tag. The X and Y coordinates detected by the cotton bale positioning inspection system were the measured values, and the positioning tests were carried out for different labels. The label position error is calculated according to the formula:

12 $$\left| {AB} \right|{ = }\sqrt {(x_{1} - x_{2} )^{2} + (y_{1} - y_{2} )^{2} } ,$$

where (x_1_, y_1_), (x_2_, y_2_) are the coordinates of two points A and B, respectively, where point A is the position of the RFID positioning tag, and B is the origin of the coordinates, The test record data is shown in Table 5.

**Table 5:** RFID tag error measurement test record.

Label number	The system monitors the label position		Manually measure label position		Monitoring distance (cm)	Actual distance (cm)	Relative error%
	X-axis (cm)	Y-axis (cm)	X axis (cm)	Y axis (cm)			
Cotton stack 1	817	127	878	121	826.81	886.30	6.7
Cotton stack 2	1369	533	1245	617	1469.10	1389.50	5.7
Cotton stack3	1969	369	1942	420	2003.28	1986.90	0.8
Cotton stack 4	407	389	425	330	523.97	538.08	2.6
Cotton stack 5	488	728	493	742	876.43	890.85	1.6
Cotton stack 6	1926	377	1931	406	1962.55	1973.22	0.5

The test showed that the RFID cotton bale positioning monitoring system, based on the test data of the RFID tag positioning information, showed that the relative error of the system monitoring was less than 6.7%. The reason for the error was that the positioning distance was based on the positioning of the tag by the base station. The accurate distance was calculated by the signal field strength. In the case of a long cotton pile, a slight error would occur. After debugging for many times, the error can be effectively improved by arranging multiple base stations, and the monitoring requirements can be met based on performance and technical indicators required by the system.

### Temperature sensor test

**Figure 18:**
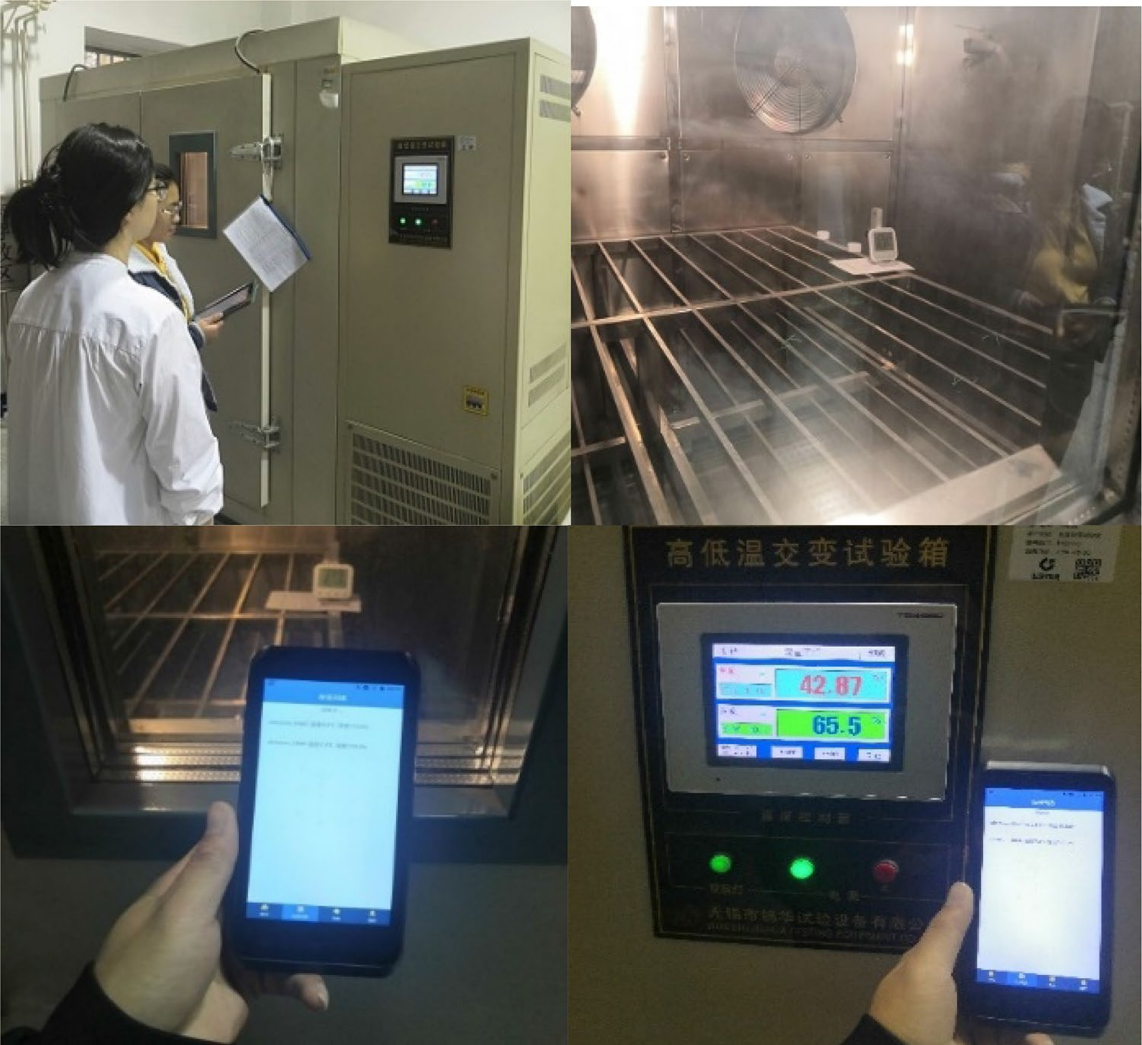
Test of temperature and humidity monitoring.

As shown in Fig. 18, in the view of the temperature and humidity monitoring situation, because the warehouse environment is not easy to change, in order to better test the temperature and humidity sensor monitoring effect, the temperature and humidity monitoring device was tested in the National Quality Inspection Center. Through monitoring the temperature and humidity in the constant temperature and humidity box, the temperature and humidity displayed on the display screen of the constant temperature and humidity box were taken as the actual value, and the value displayed on the hand-held terminal of the storage system was taken as the measurement value. The temperature and humidity value of the constant temperature and humidity box was constantly adjusted and recorded, as shown in Table 6.

**Table 6:** Temperature measurement data.

Number	Measuring temperature value /℃	Actual temperature value /℃	Error value/℃	Relative error %
1	17.87	17.30	0.57	3.3
2	16.000	15.80	0.20	1.2
3	10.60	11.00	0.40	3.6
4	9.12	9.00	0.12	1.3
5	7.37	8.00	0.62	7.7
6	5.20	5.50	0.3	5.4

Through the temperature and humidity monitoring effect test of the temperature and humidity monitoring sensor, the test results showed that the temperature monitoring error of the system was less than 8%, which met the monitoring requirements. The error was caused by the uneven temperature value in the temperature and humidity constant temperature and humidity box. By adjusting the time, the error can be effectively improved, effective monitoring can be realized within the expected index range, and temperature monitoring met the monitoring performance index requirements.

### Humidity sensor test

By adjusting the humidity value of the constant temperature and humidity box, the humidity of the temperature and humidity sensor of the system was monitored. The handheld terminal for inspection was wirelessly connected with the sensor through Bluetooth, and the data was transmitted to the bale inspection system in real-time, and the humidity of the temperature and humidity sensor was tested. When using a constant temperature and humidity box and the temperature and humidity sensor humidity monitoring comparison, testing in different environments. By adjusting the actual humidity value of the constant temperature and humidity box and the measured value monitored by the system temperature and humidity sensor, it can be concluded whether the humidity measurement of the system is normal and accurate. The test results are shown in Table 7.

**Table 7:** Temperature and humidity measurement data.

Number	Measure the temperature/°C	Actual temperature/°C	Measure the humidity /%RH	Actual humidity /%RH	Temperature relative error %	Humidity value error /%
1	17.87	17.30	− 15.80	− 15.10	3.3	4.63
2	16.00	15.80	− 28.50	− 26.90	1.2	5.94
3	10.60	11.00	34.70	33.00	3.6	5.15
4	9.12	9.00	40.50	38.70	1.3	4.65
5	7.37	8.00	51.60	50.10	7.7	3.00
6	5.20	5.50	60.50	63.50	5.4	4.72

Through the temperature and humidity monitoring effect test of temperature and humidity monitoring sensor, the test results showed that: temperature measurement error was less than 8%, and relative humidity error of the system was less than 6%. The temperature and humidity sensor monitoring device can monitor real-time temperature and humidity monitoring values, and the system ran normally and met the design requirements. The error is caused by uneven temperature and humidity in the temperature and humidity constant temperature and humidity box. By adjusting the time, effective monitoring within the range of expected indicators could be achieved and the requirements of monitoring performance indicators could be met.

## Conclusion

A scheme based on RFID warehousing intelligent inspection system was proposed in this paper, and the overall scheme design of the system was carried out, mainly including hardware design and software design. The real-time sensor technology, wireless transmission technology, and intelligent monitoring technology was applied to Cotton stack warehousing in the system, which achieved the purpose of monitoring the temperature and humidity of circulation in the cotton stack storage warehouse. Many cotton stack warehouse can be inspected in real time through hand-held terminals, and cotton package information can be uploaded to the platform in real time, which reduced the wiring cost and maintenance difficulty, also liberates the labor, and has a broad application prospect in the cotton industry.

An RFID intelligent inspection terminal was developed in the system, which integrated RFID positioning technology and wireless temperature and humidity monitoring technology in the system platform, and adopted the method of particle swarm optimization (PSO) algorithm to optimize artificial neural network (ANN) to process the system monitoring data by Gaussian filtering, and established an accurate classification model of RSSI and tag position. Through the comparison and analysis of the algorithms, the positioning error of the PSO-ANN algorithm was generally smaller than that of the ANN algorithm, that was, PSO-ANN had better positioning accuracy of cotton stacks.

Through the demonstration application and experiment of the National Quality Testing Center, the RFID cotton bale positioning inspection and temperature and humidity monitoring system were tested respectively. The test results showed that the positioning accuracy error was less than 6.7% and the temperature and humidity monitoring accuracy error was less than 8% and 7%. The system can monitor the temperature and humidity of the warehouse and the positioning information of the cotton stack, with high reliability and low cost, and effectively realize the automation, information, and intelligence of the storage management of cotton bale.
